# A Kinematic Approach for Efficient and Robust Simulation of the Cardiac Beating Motion

**DOI:** 10.1371/journal.pone.0036706

**Published:** 2012-05-30

**Authors:** Takashi Ijiri, Takashi Ashihara, Nobuyuki Umetani, Takeo Igarashi, Ryo Haraguchi, Hideo Yokota, Kazuo Nakazawa

**Affiliations:** 1 Bio-research Infrastructure Construction Team, RIKEN, Wako, Japan; 2 Department of Cardiovascular and Respiratory Medicine, Heart Rhythm Center, Shiga University of Medical Science, Otsu, Japan; 3 Department of Computer Science, The University of Tokyo, Tokyo, Japan; 4 Laboratory of Biomedical Science and Information Management, National Cerebral and Cardiovascular Center Research Institute, Suita, Japan; University Hospital of Würzburg, Germany

## Abstract

Computer simulation techniques for cardiac beating motions potentially have many applications and a broad audience. However, most existing methods require enormous computational costs and often show unstable behavior for extreme parameter sets, which interrupts smooth simulation study and make it difficult to apply them to interactive applications. To address this issue, we present an efficient and robust framework for simulating the cardiac beating motion. The global cardiac motion is generated by the accumulation of local myocardial fiber contractions. We compute such local-to-global deformations using a kinematic approach; we divide a heart mesh model into overlapping local regions, contract them independently according to fiber orientation, and compute a global shape that satisfies contracted shapes of all local regions as much as possible. A comparison between our method and a physics-based method showed that our method can generate motion very close to that of a physics-based simulation. Our kinematic method has high controllability; the simulated ventricle-wall-contraction speed can be easily adjusted to that of a real heart by controlling local contraction timing. We demonstrate that our method achieves a highly realistic beating motion of a whole heart in real time on a consumer-level computer. Our method provides an important step to bridge a gap between cardiac simulations and interactive applications.

## Introduction

The heart is a muscular organ, in which the myocardial fibers are helically aligned [1]–[3]. When the heart is beating, myocardial fibers receive an electrical signal and contract in the longitudinal direction. The accumulation of these local contractions results in a global pumping motion. Computer simulation of such motions has many potential applications, including off-line tools for identifying cardiac functions and interactive tools for supporting communications between physicians and patients, designing digital contents, or assisting education.

Many studies have been published on simulating heart motion [4]–[8]. Most of the existing cardiac-muscle models are based on Hill-type model [9]–[11]. Lin and Yin presented a multiaxial constitutive law for computing actively contracting myocardium [4]. These mechanical models were integrated with electrophysiological simulations of excitation propagation and blood flow simulation to emulate total heart behavior [5]–[8]. However, applying these methods to interactive applications is very difficult, as they usually require specific hardware (e.g., supercomputers) and off-line computational time. The main target of these models is to study cardiac mechanisms; thus, they emphasize physically precise modeling rather than computational efficiency. In contrast, our goal is to provide a simulation framework for interactive applications. Our focus is on computational efficiency and robustness.

The key idea is to employ a kinematic approach rather than physics-based approach. We compute cardiac beating motion by dividing a heart model into overlapping local regions, contracting the shapes of the local regions, and estimating a global deformation that satisfies all contracted local shapes as much as possible. When estimating the global deformation from local contractions, we apply shape-matching dynamics (SMD) method [12], [13]. SMD is a geometry-based elastic body representation used in the computer graphics field. It replaces mechanical equilibrium equations of physics-based simulation with geometric constraints and achieves high computational efficiency and unconditional robustness. As we compute organ-level beating deformation from fiber-level contractions, we call our simulation kinematic approach. We have previously presented a similar method for designing motions of soft objects such as mollusks and muscles [14]. In the present study, we extend the previous study to assess whole-heart motion. We introduce fiber direction-dependent weights to emulate anisotropic stiffness of the myocardium [15], [16] and provide tools to specify local contraction timing and myocardial fiber directions.

To evaluate the reproduction performance of our proposed method, we provide detailed comparison between our method and a physics-based method using a simplified situation. This indicated that our method achieves contracted shapes that closely resemble those of the physics-based method. Our kinematic approach has high controllability; since our method generates global motion from the accumulation of local contractions, we can easily adjust macroscopic motions of simulation results to that of observations by modifying local contraction timing. Such controllability is particularly important to reproduce observed motions. We found that the left ventricle ejection fraction (LVEF) of the simulated whole heart was very close to the average LVEF of a real heart. Since our method never requires solving equilibrium equations, it achieves high computational efficiency and robustness. With carefully tuned parameters, our method can generate realistic cardiac beating motions in real-time. To illustrate the feasibility of our real-time framework, we provide three interactive tools.

We would like to emphasize that our method does not replace existing physics-based approaches but provides an alternative. As our method is a purely kinematic simulation, it is limited to producing motions. In other words, it is difficult to obtain physical values, such as stress distributions or ventricular wall pressures, from simulation results. However, we believe our method is useful for estimating LVEF, predicting kinematic changes in response to altered fiber orientations, or generating interactive animations. Hopefully our method will expand the audience for interactive cardiac simulations.

## Methods

### Overview of the simulation framework

**Figure 1:**
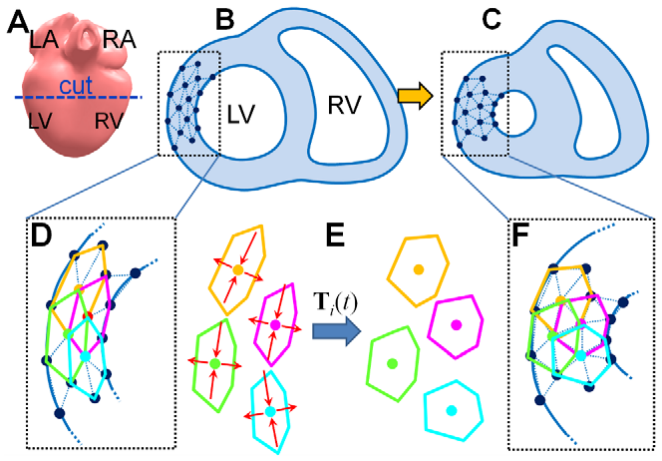
An overview of our kinematic approach. (A) Heart model. LA, RA, LV, and RV stand for left/right atrium, and left/right ventricle, respectively. (B) Target mesh model in 2D. Constructed local regions (D) are constructed along the fiber orientation so as to maintain their original volumes (E). (F, C)We deform global shape so as to satisfy the shapes of the constructed local regions as much as possible.

Figure 1 shows a two-dimensional (2D) illustration of our simulation outline. We represent a heart with a volumetric tetrahedral mesh model. In the 2D case, we consider a horizontal cross section of the heart, and the target model is represented with a 2D triangular mesh (Figure 1A and 1B). Before running a simulation, we construct a local region, *N_i_*, around each *i*-th vertex, **x** *_i_*, of the model by connecting its immediate (1-ring) neighbors (Figure 1D). The local region represents a fragment of myocardium, and neighboring local regions overlap. During each simulation step, we first deform all local regions independently, and then deform the global shape to satisfy the contracted local regions as much as possible. In Figure 1E, local regions are contracted in the circumferential direction (i.e., fiber direction) and expanded in the transmural direction. As a result, the ventricular wall thickens, and the right and left ventricles (RV and LV, respectively) shrink (Figure 1F and 1C).

A major difference between our method and traditional physics-based methods is that we replace internal force computations with local regional contractions and compute global motion using geometric constraints. This effectively avoids overshooting, an inherent problem in traditional physics-based methods, and achieves unconditional robustness.

### Contraction Function

We denote the contraction condition of the *i*-th local region, *N_i_*, at time *t*, with the contraction function **T** *_i_*(*t*). As excited myocardial fibers contract along their longitudinal direction and expand in the transverse direction, we define **T** *_i_*(*t*) as anisotropic scaling. To determine **T** *_i_*(*t*), we consider the three most important elements: local myocardial fiber direction, contraction timing, and contraction rate.

#### Myocardial fiber direction is represented with a smooth vector field, in which a single unit vector, 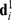, is defined at each local region, *N_i_*. Although such a vector field can be captured by using diffusion tensor magnetic resonance imaging (MRI) [17], it requires specific techniques and devices

For simulation purpose, mathematical representations are useful to represent a vector field inside a simplified LV model [3], [8]. To construct vector fields in complicated whole heart model, Takayama et al. [18] presented a sketch based interface. In this paper, we used latter two methods.

#### Contraction timing and rate are also important elements to determine contraction conditions of local regions

Some researchers have computed them using electrophysiological simulations [5]–[8], [19], [20]; however, these simulations usually require a lot of computational time. Our purpose is to achieve an interactive framework, and then we specify the contraction timing and rate by a *time-contraction curve*, which plots contraction ratio 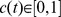 at time 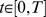, where *T* is a time cycle (Section 3 and Video S1). Given the contraction rate *c*(*t*), we compute the scaling ratio along the fiber direction as
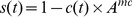
(1)where *A^mc^* 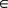
[0,1) is a *maximum contraction (MC) rate*. For example, when *A^mc^*  = 0.2, the local regions are contracted by 20% (scaled to 0.8 times) along the fiber direction when *c*(*t*) = 1.0. Note that the time-contraction curve lacks the ability to represent excitation propagation phenomena. To emulate this in an easy-to-control way, we used a phase-shift field presented in [14].

#### Contraction function

Given the fiber direction vector 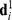 and the scaling rate 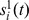 for each local region, *N_i_*, at time *t*, the contraction function **T** *_i_*(*t*)
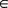
R^3×3^ is defined as an anisotropic scaling matrix,
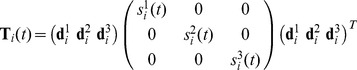
(2)where 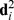 and 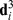 are arbitrary unit vectors orthogonal to 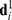, orthogonal to each other, and satisfy 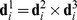. Thus, {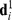, 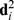, 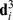} constructs a normalized orthogonal basis at *N_i_*. 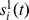, 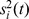, and 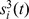 represent the scaling rate along 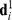 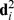, and 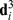, respectively. We define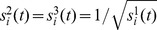 so that a scaled local region maintains its original volume.

Notice that, our simulation framework is not limited to manually designed fiber orientations and time-contraction curves; it would be easy to import fiber orientation data obtained from a real heart [17] and contraction timing obtained from excitation propagation simulations [19], [20].

### Kinematic Approach

As previously mentioned, we construct overlapping local regions, *N_i_*, at each vertex, **x** *_i_*, by connecting neighboring vertices. The contraction condition at *N_i_* at time *t* is defined by **T** *_i_*(*t*)
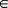
R^3×3^. For each simulation frame, we compute global motion in two steps: computing goal position **g** *_i_* and updating position **x** *_i_* and velocity **v** *_i_* of the *i*-th vertex.

#### Goal position computation

**Figure 2:**
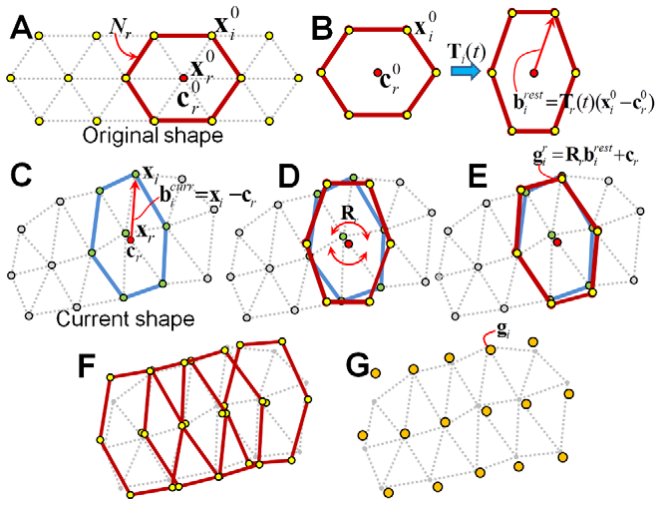
2D illustration of goal-position computation. Focus on the *r*-th region, *N_r_*. We have its undeformed original shape (A) and current deformed shape (C). The rest shape of *N_r_* is obtained by scaling the original shape (B). We translate the rest shape to fit its mass center to that of the current shape (D) and then rotate the rest shape to fit the current shape as much as possible (E). We compute the shape matching for all local regions (F) and blend the results to obtain the goal positions (G). In (F), we visualize the fitting results of only four regions.

The goal position **g** *_i_* is the desired position of the *i*-th vertex in the next simulation frame. This is obtained by computing the best-fitting rigid transformations from the rest shapes to the deformed shapes of all local regions and blending the results. Figure 2 provides a 2D illustration of the goal position computation. Let us focus on the *r*-th local region *N_r_*. The relative locations of *N_r_* rest shape vertices with respect to its mass center, 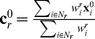, are defined as
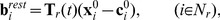
(3)where 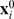 is the *i*-th vertex position of the undeformed original shape, and 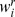 represents orientation-dependent weights (we explain this later). Note that we scale the original relative positions 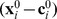 by 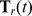. Similarly, the relative locations of the current shape vertices with respect to its mass center, 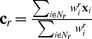, are defined as
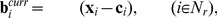
(4)where 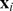 is the *i*-th vertex position of the deformed current shape. We then compute the rotation matrix 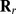 which fits the rest shape to the current deformed shape of *N_r_* by solving,
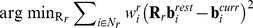
(5)


Please see [12], [14] for a detailed method to solve this minimization problem. Given the fitting rotation matrix **R** *_r_*, we can estimate the goal positions of vertices in *N_r_* as
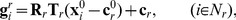
(6)


The process that computes the best-fitting rigid transformation from a rest shape to a current shape in each local region is called *shape matching*. Now, we compute shape matching for all local regions. As one vertex belongs to multiple local regions, multiple goal positions are derived for each vertex. Finally, we blend them to obtain the goal positions:
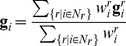
(7)


#### Update vertex positions and velocities

Given goal position **g** *_i_*, we update positions **x** *_i_* and velocities **v** *_i_* of the current simulation frame to
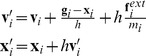
(8)where *h* is a time step, 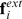 is an external force on the *i*-th vertex, and *m_i_* is the mass of the *i*-th vertex.

#### Stiffness control

**Figure 3:**
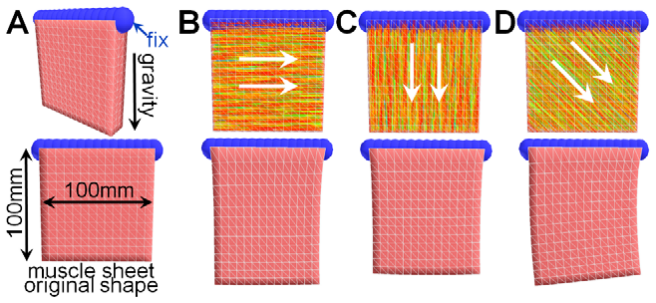
The effect of fiber-direction-dependent weights. We specified horizontal (B), vertical (C), and 45°-slanted (D) fiber orientation in a thick -sheet model (100 mm×100 mm×20 mm) (A). We then fixed the top regions and observed the resting shapes in a gravity field without activation. The resting shapes (B–D) show that the model is stiffer in the fiber direction than in the perpendicular direction. We specified gravity acceleration as 9.8 m/s^2^, time step *h* = 0.005 s, and stiffness iteration *M* = 3. Note that we used small stiffness value so as to observe large deformations.

Stiffness control is an important topic. Rivers and James [13] modified stiffness by changing the size of local regions, whereas Ijiri et al. [14] controlled stiffness by repeating the goal position computation. In our setup, the local region size cannot be changed. Because each region represents a fragment of myocardium and is linearly contracted, a large local region causes large errors. We therefore use the repetitive method [14]. We first apply shape matching to the current shape **x** *_i_* to obtain the initial goal position 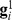. We then use 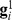 as the target shape and apply shape matching to 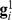 to obtain 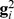. We iteratively compute 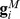 and use it as the goal position. A force applied on a vertex affects vertices farther away for a larger value of *M*, resulting in stiffer deformations. We specify *M* = 10 for all examples in this study excepting Figure 3.

#### Fiber direction dependent weights

Weighting coefficients, 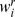, for computing the mass centers of local regions and blending shape-matching results are important in the SMD framework. Previous studies [13], [14] specified these coefficients as 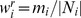, where 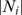 is the number of vertices in 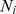. This results in isotropic elasticity. However, cardiac muscle exhibits anisotropic elastic behavior; the muscle is stiffer in the fiber direction than in the plane perpendicular to the fiber direction [11], [15], [16]. To achieve this anisotropic stiffness, we define the weights depending on the local fiber direction, 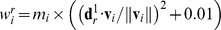, where 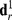 is a unit fiber direction vector at *N_r_* and 
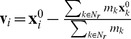
 is the location of 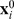 relative to the mass center of *N_r_*. That is, we place stronger weights for vertices of which the relative position is along the fiber direction. This simple modification efficiently achieves anisotropic stiffness (Figure 3).

#### Volume preservation

Although we contract local regions so that they maintain their original volumes and use them as the rest states, the total volume of the computed goal position is seldom maintained. This is because the complicated fiber orientation causes locally inconsistent contractions, and the goal position is obtained as a result of local compromise. To guarantee preservation of the total volume, we modify the goal position, similar to the volume-preserving SMD presented by Takamatsu and Kanai [21]. This method first generates a global displacement vector field from the boundary surface normal and then computes a sufficient displacement.

## Results

### Comparison with a physics-based model

**Figure 4:**
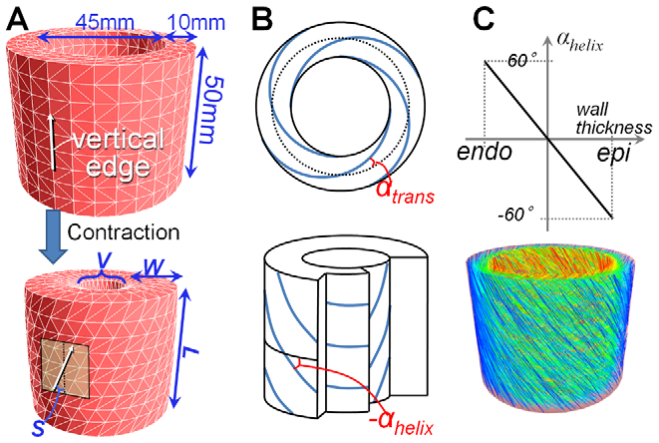
A simplified LV model. (A) A simplified LV model. (B) Two angles, *α_trans_* and *α_helix_*, denote the myocardial fiber orientation in the model. (C) The specified *α_helix_* varies linearly along the transmural direction.

**Figure 5:**
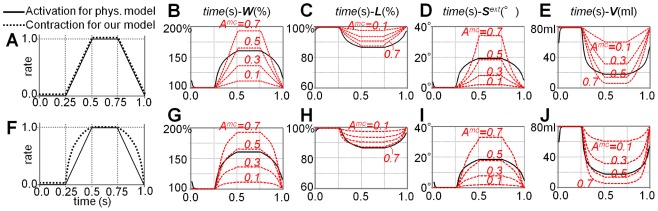
Simulation results of the simplified LV model. Simulation results computed using the physics-based method [10], [11] and our method. In the top row (A–E), we compare the two methods with the same time-activation/contraction curve (A). In the bottom row (F–J), we modify the time-contraction curve for our method (F) and compare the two methods. In (A) and (F), solid lines indicate time-activation curves for the physical method and dashed lines are time-contraction curves for our method. While (B–E) show temporal variations in the four shape measurements computed with (A), (G–J) show those computed with (F). In (B–E) and (G–J), the solid line indicates the results of the physics-based method, and the dashed red lines denote the results of our method with different MC rates.

To evaluate the reproduction performance of our method, we compared deformation simulation results generated with our kinematic and physics-based approaches. As a target for comparison, we used a 3D body extension [11] of the Zajac muscle model [9], [10]. Note that, although this is a skeletal muscle model, this primitive muscle model is very widely used and recently presented myocardial model [4] shares similar characteristics, e.g., incompressible elasticity, non-linear stress-strain relationship, and sarcomere length dependency, with the Zajac model [9], [10]. We adopted this simple but essential model to evaluate how much our kinematic approach can reproduce fundamental traits of cardiac motion. We used the following material parameters, Young's modulus, 0.02 MPa; relative density, 1.1; and maximum stress, 0.4 MPa, inferred from previous studies [10], [22]. As a simplified LV model, we prepared a thick-walled-cylinder with a 10 mm thickness, a 45 mm inner diameter, and 50 mm an axial length (Figure 4). Myocardial fiber orientation inside the model is usually denoted using two angles, *α_trans_* and *α_helix_*, which define the angle between the circumference direction and the fiber orientation on a plane perpendicular to the LV longitudinal axis and the inclination of the fiber orientation from the plane perpendicular to the LV longitudinal axis, respectively. Using this notation, we specified the fiber orientation as *α_trans_* = 0°, and *α_helix_* as linearly varying from +60° to −60° along the transmural direction [1], [3], [8] (Figure 4B and 4C). We also specified a simple time-activation curve for the physics-based method and the same shaped time-contraction curve for our method as in Figure 5A.

Both methods generate axially symmetric deformations and we measured the following four values: wall thickness, *W*; vertical length, *L*; spatial volume, *V*; and slant angle of the external surface, *S^ext^* (Figure 4A). The spatial volume represents the volume of an internal cylinder, which corresponds to the volume of the LV chamber. The slant angle is that between the cylinder axis and the vertical edges that exist on an external cylinder vertically oriented in the undeformed state. This exhibits the magnitude of twisting deformation. Figure 5B, C, D, E shows the temporal variations of the four values. The solid line corresponds to the physical model, whereas the dashed red lines correspond to the deformations computed using our method with different MC rates (i.e., *A^mc^* = 0.1, 0.3, 0.5, and 0.7).

During full activation (i.e., time 0.5–0.75), our method with *A^mc^ = 0.5* achieved values very close to those of the physics method for *W*, *V*, and *S^ext^* (Figure 5B, 5D, and 5E). The decrease ratio of *L* was less than 15% in the both methods (Figure 5C). We found a difference between the two methods in the temporal variation of the four measurements during the activation rate varying (i.e., time 0.25–0.5 and 0.75–1.0). Our method generated approximately linear variations in *W* and *L*, whereas the physics-based method resulted in nonlinear variations (Figure 5B and 5C). This nonlinearity of the physical model is caused by two major reasons; the active muscular force depends on the both activation rate and muscle elongation rate [10], and Mooney-Rivlin hyperelastic material used in [11] has a nonlinear strain-stress relationship. Whereas, our kinematic method simply translates local contractions into global deformation; the global deformations are obtained from the accumulations of local scaling and local rotation. When the influence of local scaling is dominant (i.e., influence of local rotation is small enough), temporal variations of global measurements are almost proportional to the specified time-contraction curve.

### Contraction speed fitting

The gradient of temporal variation of the global shape measurements represents contraction speed that is important clinically. For heart simulators, the ability to reproduce an observed contraction speed is very important. Using our method, the temporal changes in the simulated motion can easily be controlled by modifying the time-contraction curve based on physiological or clinical data.

As mentioned above, it can be estimated that a temporal variation of a global measurement is almost proportional to the specified time-contraction curve, when the effect of local scaling is dominant. Based on this estimation, we designed a new time-contraction curve by taking the time-*W* curve obtained by the physics-based simulation (the solid curve in Figure 5B) and normalizing its domain and range into [0,1] as in Figure 5F. We then simulated deformations with it. As a result, our method achieved temporal variations (chart shapes) very close to those of the physics simulation in *W*, *L*, and *V* (Figure 5G, 5H, and 5J). However, we found small difference in slant angle, *S^ext^* (Figure 5I). Since *S^ext^* is affected by the both local contraction and rotation, it is difficult to perfectly adjust it with our current method. Notice that the difference in *S^ext^* is small enough for our main purposes, i.e., estimating LVEFs and generating real-time animations.

### Influence of fiber orientation

To examine the influence of myocardial fiber orientation on global deformation, four different fiber orientations were specified in the simplified LV model. Then the deformation was computed using our method and the physics-based method. Because the MC rate that provides the deformation closest to that of the physics-based method depends on fiber orientation, we tested all *A^mc^* values within the interval of 0.05 for each of the four cases and selected the one that output the deformed shape closest to the physics-based simulation. We used spatial volume value as a metric for closeness.

**Figure 6:**
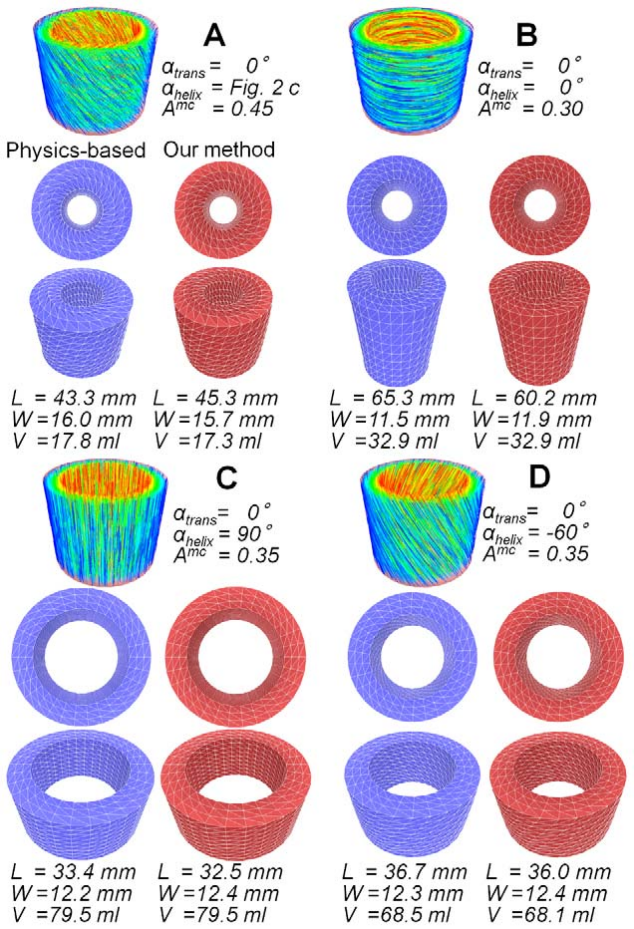
A comparison between our approach and the physics-based method using four different fiber orientations. Each panel provides the specified fiber orientation and *A^mc^* value on the top, a contracted shape with the physics-based method on the bottom left (blue), and a contracted shape with our method on the bottom right (red).

Figure 6 and Video S2 summarize the specified fiber orientations, selected MC rates, and contracted shapes with their three measurements. In all four cases, our method achieved results very close to the physics-based method. In the cases of *α_helix_* = 90° and −60° (Figure 6C and 6D), the decrease in spatial volume was very small or almost zero, meaning that the two models do not have pump ability. In the case of *α_helix_* = 0° (Figure 6B), the model was elongated in the vertical direction, which is never observed in an actual heart. Varying *α_helix_* from +60° to −60° along the transmural direction resulted in the highest decrease ratio of spatial volume, indicating that this model has potential as an efficient pump (Figure 6A).

### Whole heart model construction

**Figure 7:**
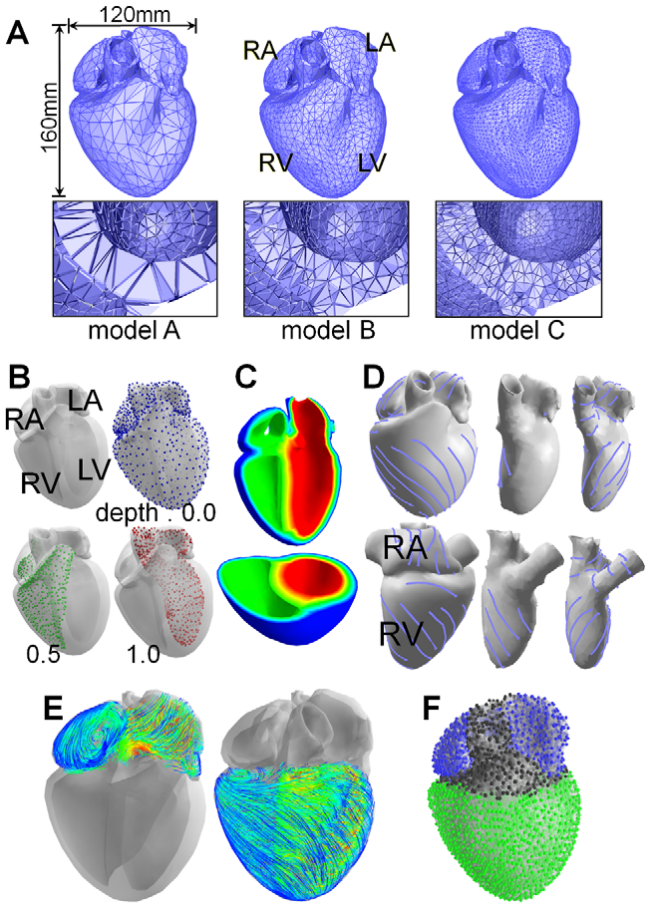
A whole heart model. (A) Three heart tetrahedral-mesh models with different subdivision levels. (B–D) Fiber orientation field construction process with the painting interface [18]. In (B), constraint points for the three regions (i.e., epicardium, RV/RA endocardium, and LV/LA endocardium) are highlighted in blue, green, and red and have depth values of 0.0, 0.5, and 1.0, respectively. In (D), light blue curves indicated the specified orientation strokes. (E) Smooth myocardial fiber orientation fields of the atrium (left) and ventricle (right) constructed. (F) Segmented local regions; atrium and ventricle regions are highlighted in blue and green.

Next, we applied our method to a whole-heart model. We constructed a tetrahedral mesh model of a whole heart from electrocardiogram-gated MRI images. We extracted the heart region from human chest images at end-diastole and generated a tetrahedral mesh from the boundary surface of the extracted region using TetGen [23]. *Model A* in Figure 7A is the obtained model. Its approximate sizes were 160 mm in the longitudinal direction and 120 mm in the lateral direction, and the wall thicknesses were approximately 11 mm (LV), 13 mm (mid-wall), and 5 mm (RV). To accurately capture the transmural variation in myocardial fiber orientation, an edge-based subdivision [24] was applied to the obtained model; we subdivided all edges of *model A* longer than 10 mm to obtain *model B* and 5 mm to obtain *model C*. Note that the LV walls of *models A*, *B*, and *C* had approximately two, three, and four vertices (layers), respectively, in the transmural direction (Figure 7A).

We manually designed a myocardial fiber orientation in the models using a two step sketching interface [18]. We first created a layer structure inside the model by placing constraint points with depth values. After placement, the depth values of all points are smoothly interpolated inside the model, and a smooth depth field is obtained (Figure 7B and C). We then draw multiple orientation strokes on each layer. The strokes represent local fiber orientation at their locations. We referred to anatomical studies on myocardial fiber orientation in ventricles [1]–[3], [17] and in atria [25] to specify the orientation strokes. The stroke orientations are interpolated inside the model resulting in smooth fiber orientation field (Figure 7D and E). We also segmented the local regions into atrial, ventricular, or excluded regions (Figure 7F).

**Figure 8:**
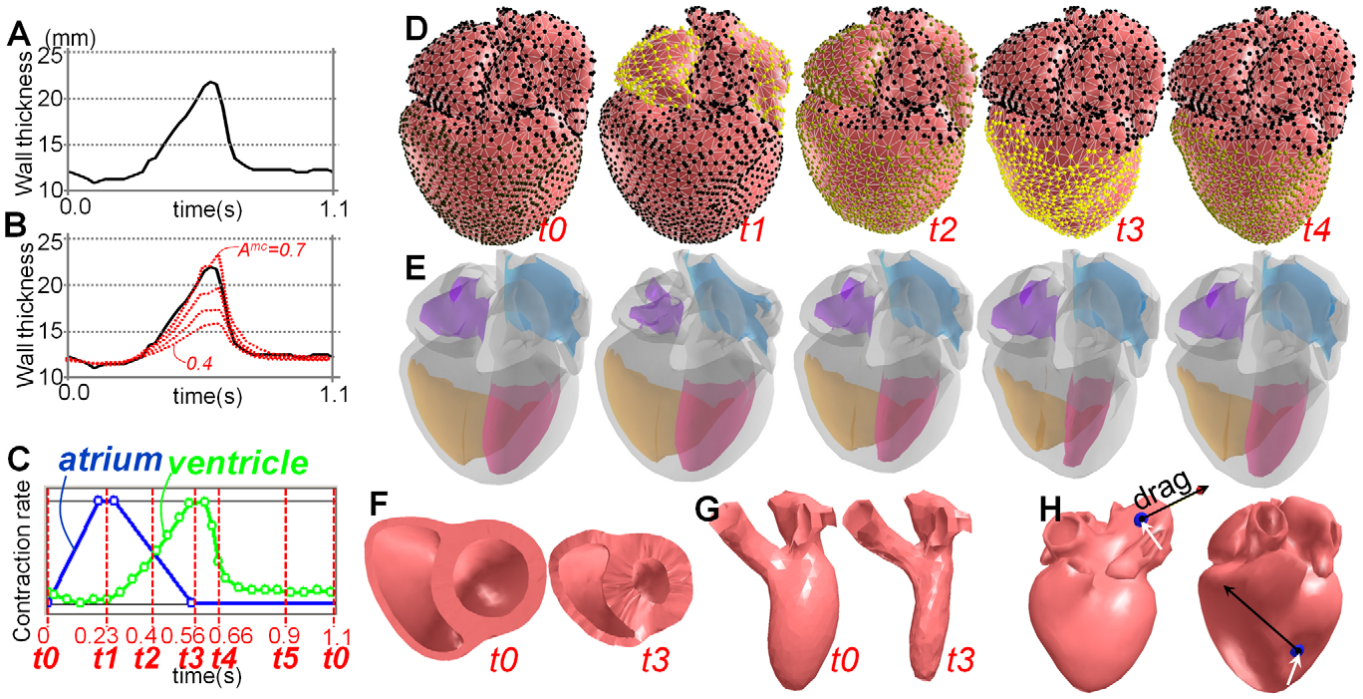
Beating motion of the whole heart model. (A) The temporal variation of wall thickness of an actual healthy heart observed by Traill et al. [26]. The four dashed red lines in (B) indicate the temporal variations of wall thickness of our simulation results with different MC rates (*A^mc^* = 0.4, 0.5, 0.6., and 0.7). (C) Specified time-contraction curve; the blue chart is for atrial regions and the green chart is for ventricular regions. (D, E) Representative frames of the whole-heart simulation with different visualization. (F–H) Three interaction tools. (F, G) Cross sections and myocardial layers of the heart at time *t0* and *t3*. (H) Direct dragging tool. White arrows indicate the grabbed point and black arrows are dragged direction.

We designed the time-contraction curve as in Figure 8C. The green chart indicates the contraction rate of local regions in the ventricle, and the blue is for those in the atrium. The time-contraction curve of the ventricle (green chart) was designed according to a real heart. A temporal variation in LV wall thickness of a real heart was observed by Traill et al. [26] (Figure 8A). We normalized this time-wall thickness curve and used it as the time-contraction curve. During *t0–t1*, the atrial regions contract, during *t1–t3* (systole), the ventricle regions contract and the atrial regions are relaxed returning to their original shapes, and during *t3–t0* (diastole), the ventricular regions are relaxed.

### Beating simulation of a whole heart

We computed the beating motions of *model B* with time cycle *T* = 1.1 s, time step *h* = 0.01 s, and different MC rates (i.e., *A^mc^* = 0.4, 0.5, 0.6, and 0.7). The dashed red lines in Figure 8B show the temporal changes of LV posterior wall thickness of the simulated beating motion. This demonstrates that our method achieves temporal changes of wall thickness that are very close to those observed in an actual heart [26]. The two rows in Figure 8D and 8E indicate representative frames of the simulated beating motion with *A^mc^* = 0.5. The top row highlights activated local regions in yellow, and the second row transparently visualizes the deformation of each chamber. Video S3 shows the simulation results in an animation.

To illustrate the feasibility of our real-time framework, we implemented three interaction tools: a cutting tool, a peeling tool, and a direct deformation tool (Figure 8F, G and Video S4). The cutting tool allows the user to temporally cut the model to observe a cross section by drawing a stroke. The peeling tool allows peeling the model and observing the motions of muscular layers. The direct dragging tool allows grabbing and dragging a vertex of the heart model. During dragging, the system adds an external force oriented to the dragged direction onto the grabbed vertex. Because our algorithm guarantees unconditional robustness, the simulation never diverges even if extremely large forces are applied.

**Figure 9:**
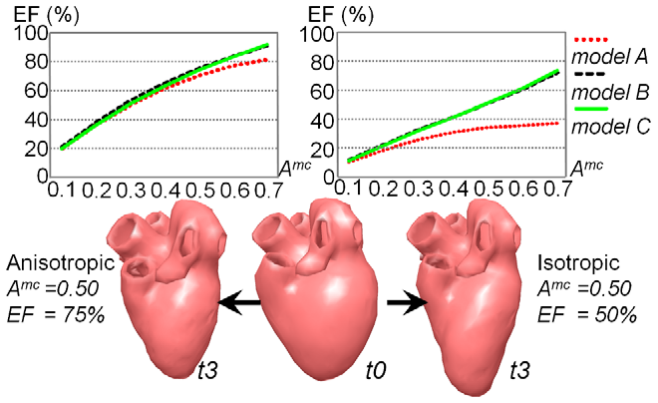
LVEFs of the simulated beating motion with respect to different MC rates. The left chart was computed with the presented fiber-direction-dependent anisotropic stiffness model, and the right chart was computed with the isotropic stiffness model. The bottom panels show the whole-hearts at end-diastole (time *t0*) and end-systole (time *t3*).

We next observed LVEF, which is a common parameter for evaluating cardiac function, to evaluate further the resulting motion quantitatively. We simulated the beating motions of *models A*, *B*, and *C* using the same setup as shown in Figure 8. The two charts in Figure 9 indicate the LVEF of the three models with respect to different MC rates. While we used the anisotropic stiffness model to obtain the left chart, the right was computed using the traditional isotropic stiffness model [13], [14].

Our orientation-dependent stiffness model generated a higher LVEF than the isotropic stiffness model. This can be explained as follows. All ventricular regions contract along the fiber direction and expand in the orthogonal direction. Our anisotropic model places higher weights on the contraction along the fiber direction and lower weights on the orthogonal expansion, whereas the isotropic model deals with the contraction and expansion fairly. As a result, the anisotropic model causes a strange vertical elongation (Figure 9, bottom right). Also, no significant difference in LVEF with respect to model resolution was observed in the anisotropic stiffness model.

## Discussion

### Evaluations of simulated motion

We compared the motion simulated by our approach and the physics-based method (Figures 5 and 6) and demonstrated that our kinematic method achieved contracted shapes that were very close to those of the physics-based method.

We applied our method to whole-heart models. Figure 8 shows that our method achieved beating motion with a physical appearance comparably close to real heart motion. The local contraction ratio of actual healthy hearts is approximately 15% and the LVEF is 60–80% [27]. Meanwhile, in our framework, the MC rate, *A^mc^*, represents an ideal local contraction rate and an actual local contraction rate is usually smaller. This is because the complicated fiber orientation causes inconsistent local contraction, and the global shape is obtained with local compromises. Taking this into account, the LVEF of our simulated beating motion of anisotropic stiffness model in Figure 9 (∼75%) was within the normal range of the LVEF of actual heart (60–80%). This suggests that our kinematic approach can recreate physiologically consistent heart contraction.

Since our kinematic approach directly compute global deformation from the accumulations of regional fiber-level contractions, it allows temporal variation of global shape measurements to be easily controlled by modifying the time-contraction curve so that its shape is proportional to the desired temporal variation curve (Figures 5 and 8). This controllability is particularly useful for reproducing observed motions. A cardiac simulation method with such a high controllability has never been achieved before.

### Performance

Table 1 shows the number of vertices of the three heart models and the total time taken to compute a single simulation frame. The number of vertices is equivalent to the number of local regions. Timing was generated on an Intel Core i7 3.33-GHz machine. Our system achieved an interactive frame rate even for the finest model. Table 1 also provides simulation times for the simple LV model (Figure 4) computed using our method and the physics-based method [10], [11]. Since it is possible to accelerate the physics-based method retaining similar results by reducing the degrees of freedom, we provide its computational time with a coarser discretized LV model (bottom of Table 1). The coarser model was created by halving the number of elements in the axial and circumferential directions. These results indicate that our method outperforms the physics-based method in computational time. Notice that this timing is limited to our specific implementation and do not fairly compare the kinematic and physics-based methods. However, we believe that our kinematic method is more efficient since it does not solve equilibrium equations and is more suitable for real-time applications. Also, further acceleration of our method would be possible using GP-GPU techniques [28], [29].

**Table 1:** Simulation Performance.

	No. of vertices	Time/frame (ms)
*Model A*	2479	7.3
*Model B*	7116	24.2
*Model C*	28685	110.7
*Simple LV*(*Fig. 4*) (*our method*)	1200	4.1
*Simple LV*(*Fig. 4*) (*physics-based method*)	1200	554.2
*Coarser discretized Simple LV* (*Fig. 4*) (*physics-based method*)	300	118.3

Time/frame indicates an average time to compute a single simulation frame.

### Potential applications

The biggest advantage of our method is its computational efficiency and robustness. Because our simulator can run on a consumer-level PC, it is accessible to many people with little knowledge of computer-science and would be a good tool for understanding the heart. If it were integrated into electronic medical-chart systems, the real-time beating animation generated by our system would help patients to understand their disease conditions and expected recoveries. Similarly, education, digital content creation, and surgery simulators are good potential applications of our system.

Although our method is not based on a physical model, little difference was found between the physics-based method and our approach using a simplified LV model with the same fiber orientation. We believe that our method can provide a useful tool for studying the influence of specific fiber orientation on the LVEF. If the mechanical changes of a patient with myocardial infarction were integrated into our method, the LVEF of the infarcted heart could be estimated immediately, and the abnormal LV wall motion could be observed in real time on a consumer-level PC.

### Limitations and Future work

Since our method induces global motion not by inner forces but by local contractions, at present it is unable to observe some physical values, such as stress distributions or ventricular wall pressures. It is also difficult to estimate the relationship between macroscopic deformation speed and internal muscular forces. The quantitative relationship between the SMD and various physics-based methods has not yet reported, and its detailed analysis remains as our future work. Another future work includes integrating electrophysiological simulations or fluid simulations into our method and comparing our simulation results with the motions of actual hearts captured by four-dimensional imaging devices. We also intend to tackle an inverse elastic body kinematics problem, in which local contraction conditions will be estimated from observed global deformation.

### Conclusions

We have presented a kinematic method for efficiently and robustly simulating the beating motion of the heart in real time on a consumer-level PC. Our method computes motion in two steps: contracting local regions along the fiber direction and estimating the global shape that best satisfies all contracted local regions by SMD. By introducing fiber-direction-dependent weights into SMD, we can emulate the anisotropic stiffness of the myocardium. A comparison between our method and a physics-based method [10], [11] indicated that both methods generated very similar beating motions. Our method achieved a highly realistic beating motion with a LVEF comparable to that of an actual heart. We found that our method has high controllability; it allowed us to easily adjust the contraction speeds of the simulated motion to observed data by modifying the time-contraction curve. Finally, we believe that our method provides an important step to bridge a gap between the unwieldy heart contraction simulations and the user-friendly interactive applications. Hopefully, this method will be used in various interactive applications and enhance the value of cardiac simulations.

## Supporting Information

Video S1 **A basic interface of our prototype system.** This video shows a basic interface for modifying local contraction rate and timing, and our real-time simulation result.
(WMV)

Video S2 **Comparison between our method and a physics-based method.** This video shows simulation results generated with our method and the physics based method side-by-side.
(WMV)

Video S3 **Left ventricle ejection fraction of a simulated whole heart.** This video shows simulated whole heart motions with three different maximum contraction rates.
(WMV)

Video S4 **Three interaction tools.** This video shows our three interaction tools, a cutting tool, a peeling tool, and a direct deformation tool.
(WMV)
